# Mechanical Behavior of Plaster Composites Based on Rubber Particles from End-of-Life Tires Reinforced with Carbon Fibers

**DOI:** 10.3390/ma14143979

**Published:** 2021-07-16

**Authors:** Rafael Vicente Lozano-Díez, Óscar López-Zaldívar, Sofía Herrero-del-Cura, Pablo Luís Mayor-Lobo, Francisco Hernández-Olivares

**Affiliations:** 1 Departamento de Tecnología de la Edificación, Escuela Técnica Superior de Edificación, Universidad Politécnica de Madrid, 28040 Madrid, Spain; rafaelvicente.lozano@upm.es (R.V.L.-D.); sofia.herrero@upm.es (S.H.-d.-C.); pabloluis.mayor@upm.es (P.L.M.-L.); 2 Departamento de Construcción y Tecnología Arquitectónicas, Escuela Técnica Superior de Arquitectura, Universidad Politécnica de Madrid, 28040 Madrid, Spain; f.hernandez@upm.es

**Keywords:** gypsum, plaster, ELT rubber, carbon fibers, composites

## Abstract

The principal objective of this research project is the disposal of end-of-life tire rubber waste and its incorporation in gypsum composites. As a continuation of previous projects, which established a reduction in the mechanical properties of the resulting products, the behavior of these composites is analyzed with the incorporation of carbon fibers. The density, Shore C hardness, flexural strength, compressive strength, dynamic modulus of elasticity, strength–strain curves, toughness and resistance values and microstructure of the material are studied and compared. The results obtained show a significant increase in the mechanical tensile strength of all of the samples containing fibers. The moduli of elasticity results show a decrease in rigidity and increase in toughness and resistance of the material produced by incorporating the fibers. An optimum dosage of a water/gypsum ratio of 0.6 and incorporation of 1.5% carbon fibers is proposed. This lightweight material, which offers a high mechanical performance, features characteristics which are suitable for large prefabricated building elements in the form of panels or boards.

## 1. Introduction

The growing need to dispose of waste in modern society, due to the significant increase in the rate at which it is generated, has made the reuse and recycling of materials a primary objective in recent years. Numerous examples can be found of studies which analyze different kinds of waste which can be used as a beneficial alternative to direct disposal [1,2,3]. Some types of harmful industrial waste can be reused, recycled and incorporated as raw materials in various areas of the building construction process, ultimately protecting the environment from possible risks [4,5,6,7].

End-of-life tires (ELTs), are a type of waste which, according to the European Council Directive 99/31/EC [8] on waste disposal, cannot be disposed of in landfills due to the fire hazard they present on account of their high calorific value, the fact that the densification of tires is difficult and due to the ease with which they accumulate gases and leachates, among other factors. It is calculated that around 1.5 billion tons of tires are generated annually worldwide [9]. This waste and the accumulation of tires, given that they are composed of a non-biodegradable material, have become an international environmental concern [9]. However, in recent years, detailed research has been carried out related to the sustainability process of used tires through recycling, since this material has high potential as a source of valuable raw materials. Such research projects are aimed at developing different procedures for reusing and recycling tires. Among the various lines of research looked that have been at, the most common are those related to recycling tire rubber, transforming it into a granulated product, or into powder, featuring different gradings [10,11]. One variation of these research projects uses the aforementioned rubber in the form of fibers from tire retreading in different volumetric fractions, previously classified in different sizes [12]. Likewise, research projects linked to the recycling and recovery of both textile and metallic fibers are of great interest.

Where construction products which incorporate this waste are concerned, as part of the first of the lines of research mentioned, numerous researchers have worked on incorporating granulated or powdered ELT rubber into the hydraulic binders typically used in construction. Their use in cement mortars is particularly widespread [13], with studies extending to aspects such as the effect of the size of the rubber particles, the substitution percentage and the possible dosages [14], or to analyzing the behavior of the different geometric shapes of the construction elements [15]. One of the applications that is being actively researched is the use of rubber from end-of-life tires as a partial substitute for conventional aggregates in concrete applications [16,17,18] and, to a lesser extent, in gypsum compounds [19]. In the literature reviewed, a decrease in density is observed, resulting in lighter materials [20,21], together with reduced rigidity [22], which gives rise to a greater capacity to absorb energy produced through deformation with the corresponding improvement in acoustic behavior [23] and reduction in cracking [24]. Modifications in the material’s microstructure are also observed [25] along with improvements in thermal behavior [26] and fire performance [27]. Nevertheless, the literature reviewed highlights the progressive loss of both flexural and compressive strength, as the proportion of ELT rubber is increased [28,29,30].

Additionally, various projects show how the incorporation of fibers in different binders is a widespread practice for improving the mechanical properties of the material, increasing the flexural tensile strength, the toughness or impact strength. The fibers used include polypropylene [31], basalt [32], glass [33], polyolefin [34,35] and mineral wool fibers [36]. Other studies have analyzed the use of carbon fibers in gypsum composites in aspects such as the reduction in electrical resistivity that enhances electromagnetic interference shielding effectiveness [37]. In other cases, the noise absorption capacity of composites consisting of gypsum boards and a carbon fiber membrane has been studied [38]. Finally, the results of expanded graphite/paraffin gypsum-based composites with the addition of 3 mm long carbon fiber [39] concluded that carbon fiber is a promising candidate for enhancing the thermal and mechanical properties of structural–functional integrated building material. Carbon fiber has also been used to reinforce cement-based composites due to its high tensile strength [40,41,42]. They explained that carbon fiber could restrain the growth of cracks and it bonds well with hydration products.

On the other hand, it is perceived that the rigidity of certain binder materials has negative aspects which are reflected in some of their properties, such as their limited thermal and sound insulation or lower impact strength, and as such have a lower capacity to prevent cracking. The incorporation of rubber from end-of-life tires (ELTs) has been used as a procedure to reduce the rigidity of this type of material, as well as recover a material that is difficult to dispose of [43,44]. Likewise, the influence of fiber length and of the percentage of fibers added is highlighted, as well as the degree of dispersion, although in all of the research projects analyzed, no studies concerning the effects of incorporating fibers composed of other materials into gypsum–ELT rubber composites were found.

Taking into account the preceding analysis, the aim of this research is to develop a lightweight material with improved elastic and mechanical properties. This decrease in density and the improvement in the elastic properties is produced by the incorporation of rubber from the recycling of end-of-life tires (ELTs) in powder form [19]. Likewise, the improvement in the mechanical properties is intended by the incorporation of carbon fibers as an application of the patent developed by the research team [45].

## 2. Materials and Methods

### 2.1. Materials

The materials used to prepare the gypsum composites are as follows: 

E-35 Plaster, belonging to the company ALGÍSS URALITA (Madrid, Spain). According to the manufacturer’s technical report, its technical characteristics are as follows (Table 1):

#### 2.1.1. End-of-Life Tire (ELT) Rubber Waste 

**Figure 1:**
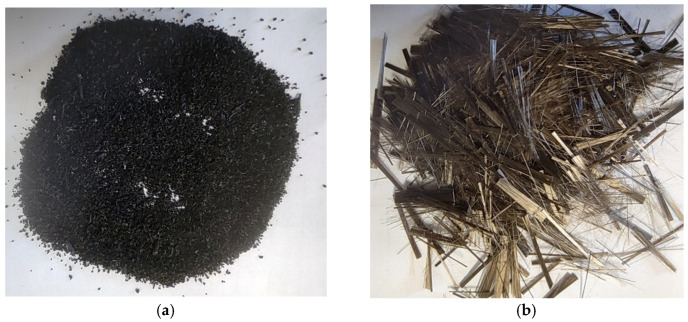
Physical appearance of the materials used. Powdered rubber (**a**) and carbon fibers (**b**).

Powdered rubber is produced by mechanically crushing end-of-life tires (ELTs) in an ambient atmosphere supplied by the ELT treatment plant which the company VALORIZA environmental services has in Chiloeches (Guadalajara, Spain). The physical properties and chemical analysis, as set out in the product technical details, are shown in Table 2. The granulometric analysis of the powdered product used is set out in Table 3, whilst the physical appearance of the material can be seen in Figure 1a.

#### 2.1.2. Carbon Fibers

The properties of the selected fibers are shown in Table 4 and their appearance in Figure 1b. The carbon fiber C-BI-AX-360 was supplied by the company Mapei.

### 2.2. Dosages

Powdered ELT rubber was used to produce the samples in a fixed proportion of 5% by weight, in relation to the weight of the plaster. The 5% percentage was chosen as a consequence of the results obtained in preliminary studies in which the incorporation of rubber in greater proportions caused significant loss of strength. 

The proportion of carbon fiber added was varied in proportions by weights of 1%, 1.5% and 2%, in relation to the weight of the plaster.

Two different groups of series were produced, modifying the water and solid phase ratio (w/g_1_), this being 0.6 in one group and 0.8 in the other. Previous studies involving a vibrating table testing and following the procedure set out in UNE-EN 13279-2:2014 [46] showed that the incorporation of rubber requires a larger initial quantity of water, and, in addition, the use of fibers lead to reduced workability.

When naming the samples, 0.6 or 0.8 was used to indicate the w/g_1_ ratio, followed by R5 to reflect the rubber content and finally the letter C to indicate the proportion of carbon along with the specific amount added. Table 5 shows the name and composition of the mixes studied.

Where the mixing procedure is concerned, the UNE-EN-13279-2: 2014 [46] standard was followed, involving dry mixing the gypsum and rubber together first and then adding the carbon fibers, then stirring the mixture manually until the fiber was uniformly distributed. This procedure was used to produce three prismatic samples measuring 4 cm × 4 cm × 16 cm for each series for the physical and mechanical characterization of the samples.

### 2.3. Tests

The physical and mechanical tests were performed in accordance with the procedures set out in the relevant UNE standards concerning gypsum and plaster. They established: dry density, flexural strength and compressive strength (UNE EN 13279-1 [47] and UNE EN 13279-2 [46]).

Portable ultrasound measuring equipment, (Ultrasonic tester E-46 IEP, PCE Ibérica, S.L. Albacete, Spain), was used in order to calculate the propagation speed. The readings for each test piece were taken in a longitudinal direction. The procedure followed in the test is based on the guidelines set out in the European standard UNE EN 12504-4 2006 [48].

**Figure 2:**
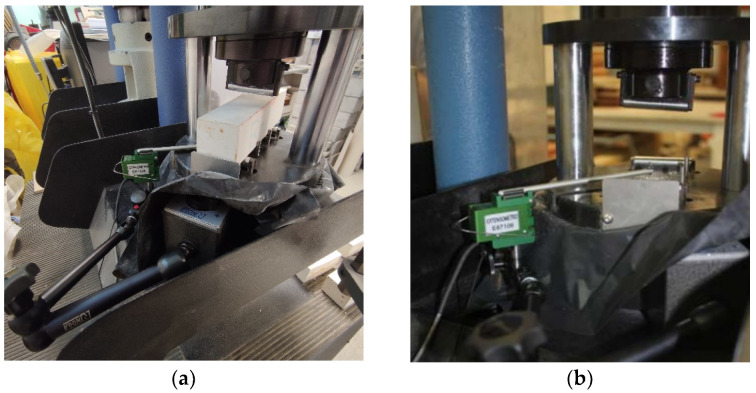
Strain gauge for measuring the deflection. Instrument calibration (**a**) and use with test pieces (**b**).

Resistance and toughness values were measured with the same machine used to obtain stress–strain graphs. In this case, a device was added to measure the deflection produced as a function of the added load. This is a strain gauge that is mounted on an articulated stand which is fastened to the test machine and remains in contact with the center of the underside of the test piece (Figure 2). 

## 3. Results and Discussion

The mixes which only contained powdered rubber were highly workable in the case of both w/g_1_ ratios. The incorporation of carbon fibers in proportions of 1% had no notable effect in this respect. However, on increasing the proportion of carbon fibers, mixing became more difficult. This may be due to a considerable loss of fluidity in the mix, it being necessary to vibrate it in order to prevent the formation of voids. The mixing difficulties occurred in the test pieces made with proportions of fiber of 2% and w/g1 ratios of 0.6.

Table 6 displays the Shore C hardness, density, flexural strength, compressive strength and dynamic moduli of elasticity values obtained for all of the series produced.

### 3.1. Shore C Hardness

**Figure 3:**
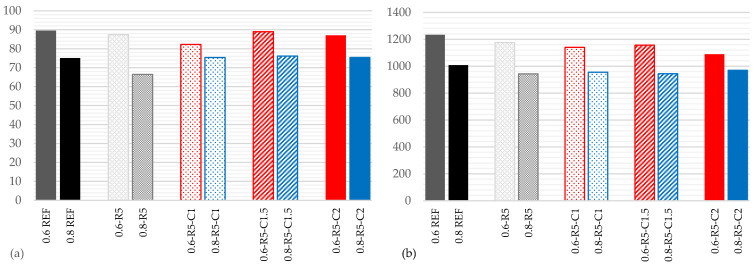
Results for Shore C hardness (**a**) and densities in Kg/m^3^ (**b**).

Figure 3a shows the Shore C hardness results. In comparison with the reference series with a w/g_1_ ratio of 0.6, they are slightly lower for all of the samples. In the series with a w/g_1_ ratio of 0.8, the decrease is greater in the sample that only contains rubber, but the Shore C hardness values recover in the series containing carbon fibers, and results similar to those of the reference series are obtained.

The incorporation of carbon fibers offsets the decrease in Shore C hardness caused by the inclusion of rubber. The differences in values are not considered to be relevant in any of the samples.

### 3.2. Density

A reduction in density of between 5% and 6% is observed in Figure 3b, in the samples containing only rubber, for w/g_1_ ratios of both 0.6 and 0.8. These values remain very similar when carbon fibers are incorporated, reducing slightly for w/g_1_ ratios of 0.8 in the mixes containing carbon fiber.

Although the values are not very relevant, the reduction in density in all of the samples compared to the reference samples stands out.

### 3.3. Flexural Strength

**Figure 4:**
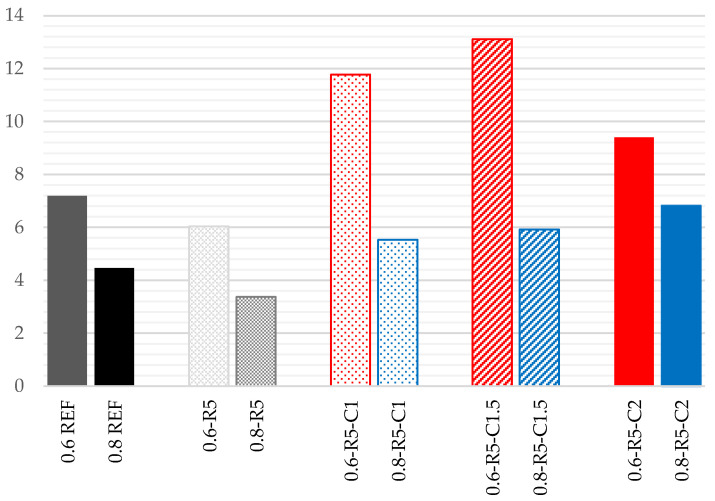
Results for flexural strength (MPa).

The results of the test pieces subjected to flexural strength testing (Figure 4) show decreases in strength compared to the reference series, in the samples only containing ELT rubber. In the series where the w/g_1_ ratio is 0.6 there is a 16% reduction, and in the series where the w/g_1_ ratio is 0.8 there is a 24% reduction. 

However, the incorporation of carbon fibers completely reverses this tendency, obtaining highly notable values which exceed the strength of the reference series. For w/g_1_ ratios of 0.8, the increases in relation to the reference series are 23% for incorporations of 1% fiber, 32% for 1.5% fiber and 53% in the case of additions of 2% carbon fiber. For w/g_1_ ratios of 0.6, the increase is much higher, it being 64% in the case of incorporations of 1% fiber and 82% in those samples which contain 1.5% carbon fiber.

Meanwhile, the increase is less when 2% fiber is added, reaching only 30% when compared to the reference series. This change in tendency may be due to the reduced workability previously mentioned. Furthermore, these results confirm previous studies on cement mortars [42] in which the addition of carbon fibers enhances their mechanical properties due to their ability of restraining the growth of microcracks and absorbing energy. However, the enhancement carbon fiber provides to cement mortar will decrease when its content is too high (2%). These amounts cause the fibers to clump together, resulting in larger air voids or gaps.

### 3.4. Compressive Strength

**Figure 5:**
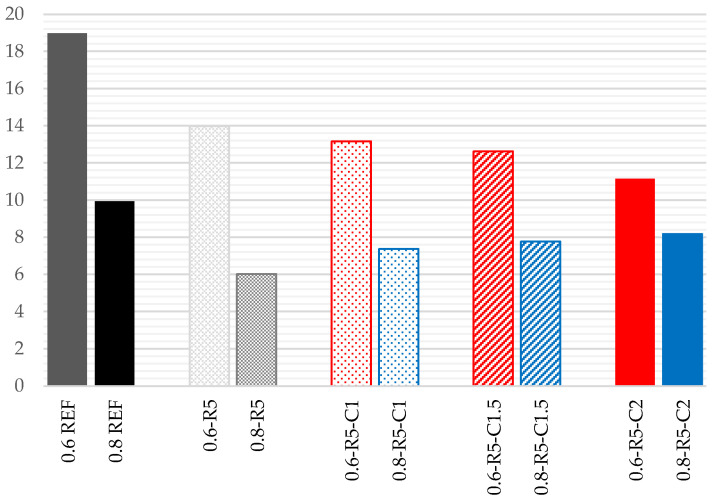
Results for compressive strength (MPa).

The significant decrease in compressive strength due to the incorporation of ELT rubber had already been established in previous projects [13,20,26]. Although the rubber particles bond to the gypsum, they act as voids that interfere with the crystalline structure of the gypsum. In addition, the presence of micropores around the rubber aggregates was observed. Both issues lead to a reduction in strength. In this study, in which the composites only contain 5% rubber, a significant reduction in compressive strength also occurs (Figure 5). This reduction amounts to 27% in those composites with a w/g_1_ ratio of 0.6, and to 40% in those composites with a w/g_1_ ratio of 0.8.

The incorporation of carbon fibers offsets this reduction slightly when the w/g_1_ ratio is 0.8, and progressively increases as the proportion of fiber is increased. As such, the loss of strength is reduced to 17% when compared to the reference series, when the percentage of carbon fiber is 2%.

However, in the composites with a w/g_1_ ratio of 0.6, the decrease in compressive strength progressively becomes more accentuated as the proportion of carbon fiber is increased, reaching 41% for fiber proportions of 2%. In the compounds with a w/g_1_ ratio of 0.8, the greater plasticity of the mixture allows a larger dispersion of the fibers, which are covered by plaster and increase the compressive strength. On the other hand, with w/g_1_ ratios of 0.6, the fibers clump together due to the lower plasticity of the mixture, creating air voids among them and reducing the compressive strength. As previously verified by Han et al. [42], in cement mortars, the reduction in compressive strength is larger as the percentage of fibers increases.

### 3.5. Young’s Modulus

**Figure 6:**
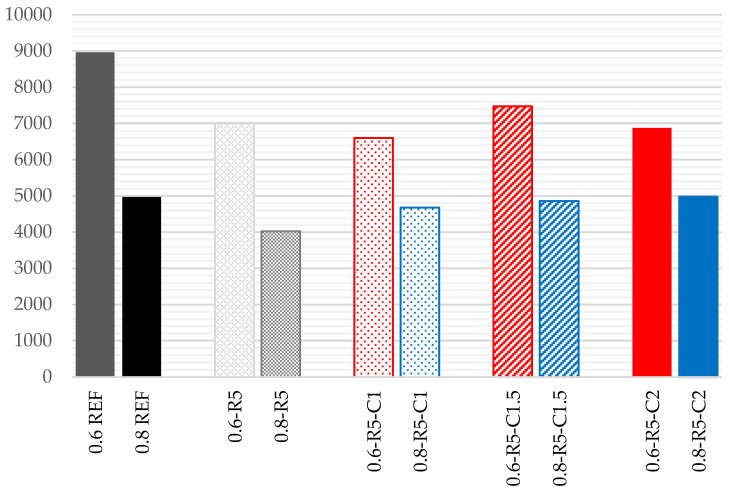
Young’s modulus (MPa).

In the graph shown in Figure 6, it is observed that the ultrasonic speed is reduced, in comparison to the reference series, with the incorporation of ELT rubber, and progressively increases as carbon fiber is added, reaching values which are similar to reference sample 0.8-R5-C2. The sample which incorporates 2% carbon fibers in w/g_1_ ratios of 0.6 breaks this tendency, producing a lower modulus of elasticity value. This result confirms the possible formation of voids or larger gaps in the sample which will lead to a decrease in ultrasonic speed.

### 3.6. Stress–Strain

**Figure 7:**
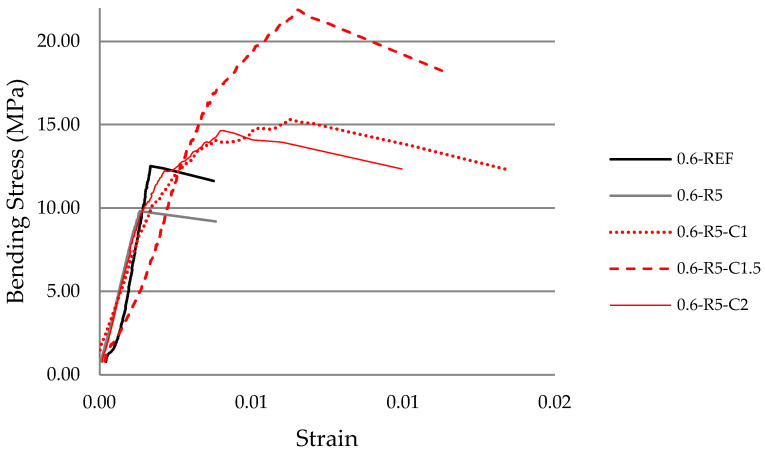
Experimental stress–strain relationship in bending tests on prismatic samples of the different mortar mixes (w/g_1_ 0.6).

**Figure 8:**
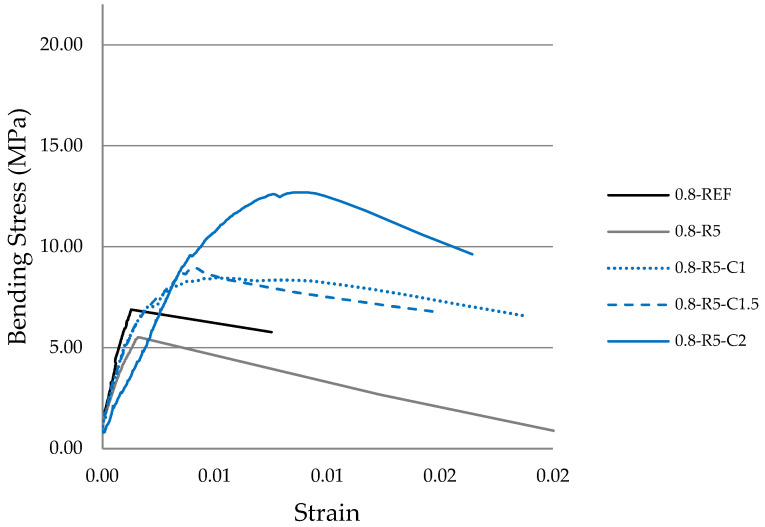
Experimental stress–strain relationship in bending tests on prismatic samples of the different mortar mixes (w/g_1_ 0.8).

The stress–strain graphs (Figure 7 and Figure 8) were produced using the deflection values recorded depending on the load applied for each of the series analyzed.

The test pieces containing carbon fibers display a significant increase in flexural strength (modulus of rupture, MOR) and in the toughness and resistance parameters (Table 7).

In the graphs reflecting w/g_1_ ratios of 0.6 (Figure 7), the slopes of the curves for the reference sample and that containing a 0.5 proportion of rubber are very similar and, in addition, a brittle fracture can be observed in both samples when the maximum stress is reached. As such, the small amount of rubber incorporated does not appear to improve the elastic behavior. However, the curves associated with the series containing carbon fiber feature slopes which rise gently until the maximum stress is reached thereby demonstrating better elastic behavior.

In the graphs reflecting w/g_1_ ratios of 0.8 (Figure 8) a difference can be observed in the slope of the curve for the sample containing rubber in a proportion of 5% when it is compared to the reference sample. The series containing carbon fiber reached the maximum stress, which is higher in all of them than in the reference sample, with curves with much gentler slopes. The sample containing 1% carbon fiber is worth a particular mention since, once the maximum stress is reached, it remains virtually horizontal for a long period of time, with the plastic branch having a very low slope.

In view of the graphs obtained, we can confirm that the samples which only contain rubber display a decrease in the maximum stress reached in comparison to the reference samples, but they show slightly better elastic behavior.

The samples containing carbon fibers reach much higher maximum stresses than the reference sample and, furthermore, once this maximum tension has been reached, in what is the plastic period of the curves, the samples remain bound together by the carbon fibers, maintaining a residual load which, in some cases, is considerable. In other words, the fibers collaborate with the plaster in a gradual rupture, increasing the capacity to absorb strain energy.

The composites mixed with a w/g_1_ ratio of 0.8 produce a stress–strain graph that is more in line with the expected results. It is true that in those samples containing only rubber, the modulus of resistance (MOR) decreases, but with the addition of carbon fibers in the different proportions mentioned above, this progressively increases. Meanwhile, with w/g_1_ ratios of 0.6, a uniform tendency is observed except in those test pieces containing a larger quantity of carbon fibers. The previously detailed issues with this type of dosage are once again confirmed.

The toughness of the composites analyzed in this research (Table 7) was calculated using the defined area below the stress–strain curves from flexural testing (Figure 7 and Figure 8). Likewise, the resistance (Table 7) was calculated as the area in the bending stress–strain graph below the linear behavior in the elastic range. Both results are expressed in MJ/m^3^. It is noted that the incorporation of carbon fibers increases the toughness of the composite material for both w/g_1_ ratios. However, the resistance gradually increases in the compounds with a w/g_1_ ratio of 0.6, and displays similar value to those of the reference sample except for samples containing 2% carbon fiber.

### 3.7. Microstructure

**Figure 9:**
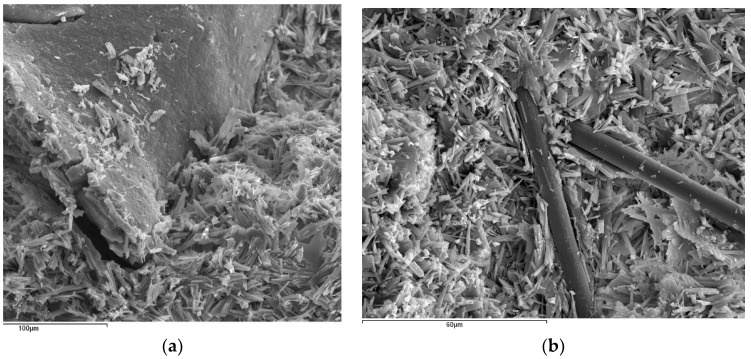

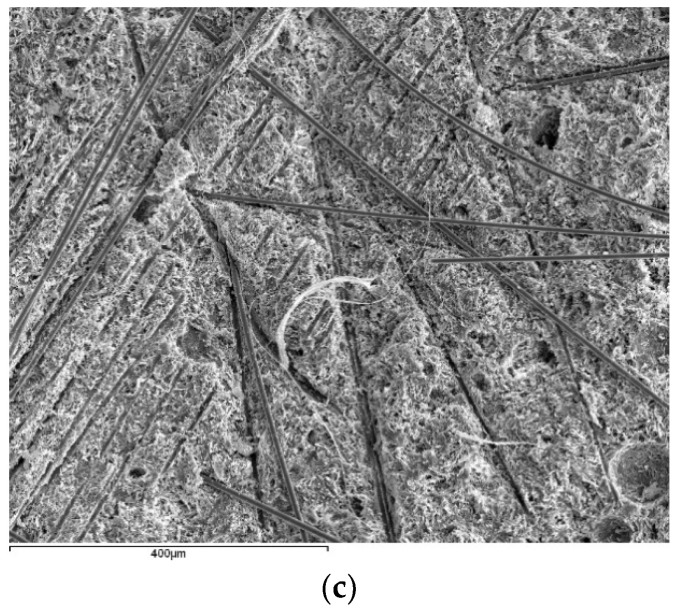
Scanning electron microscope (SEM) images of the composites: (**a**) transition area between the rubber and the plaster matrix (500×-20kV-S.E.); (**b**) transition area between the carbon fibers and the plaster matrix (1000×-20kV-SE); (**c**) grid structure of the carbon fibers (150×-20kV-S.E.).

The transition areas between the rubber and carbon fibers and the plaster matrix (Figure 9) were studied using a JEOL JSM-820 scanning electron microscope (SEM) (Research Support Center. Universidad Complutense. Madrid, Spain). The software used by the equipment for the acquisition, processing and evaluation of the analyses was EDX Oxford ISIS-Link. The carbon fibers were distributed using a random grid structure with a high level of dispersion, although a certain degree of clumping can also be observed in some areas (Figure 9c), particularly in the samples containing 2% carbon fiber and a w/g_1_ ratio of 0.6, with this having a negative effect on the workability of the fresh product. Likewise, this structure can restrict the segregation of the mix during mixing, reducing the plasticity of the material. Despite the fact that the fibers display a certain rigidity in the majority of the cases analyzed, in the visual analysis of the SEM photographs, no microcracks are detected at their ends.

The rubber particles and carbon fibers are found to be totally covered in gypsum crystals (Figure 9a,b). Neither the morphology of the hydration products nor their size is affected by the incorporation of ELT waste and the aforementioned fibers. They display reasonable uniformity in the transition areas, creating stable mechanical cohesion with no chemical reactions on the interface to justify the increase in flexural strength of the resulting material.

## 4. Conclusions

In accordance with the results obtained, the following conclusions may be drawn:

The addition of rubber reduces the Shore C hardness, although the incorporation of carbon fibers offsets this decrease, obtaining similar values to those of the reference series. 

The incorporation of rubber produces a reduction in density which remains when fibers are included in the composites. The reduction percentage ranges between 5% and 6% in comparison to the values obtained for the reference samples.

The addition of rubber in proportions of 5% significantly reduces the compressive strength for both w/g_1_ ratios studied. The incorporation of carbon fibers in the w/g_1_ ratio of 0.8 results in a gradual increase of compressive strength without fully offsetting the loss caused by the rubber.

The increase in flexural strength in samples which incorporate carbon fibers when compared to the reference series is particularly noteworthy, with these achieving increases of 30% for w/g_1_ ratios of 0.6, when 2% fiber is incorporated, particularly if we consider the significant reduction in flexural strength caused by incorporating rubber (16% in w/g_1_ ratios of 0.6).

The toughness, resistance and modulus of resistance (MOR) increase in all of the dosages incorporating carbon fibers, exceeding their respective control dosages.

It may be concluded that flexural strength significantly increases with a minimal incorporation of carbon fibers and there is a rising tendency when the proportion of fibers is increased. At water/gypsum ratios of 0.6, the flexural strength increases by 95% for 1% carbon fiber incorporations and by 117% for 1.5%. Similarly, at water/gypsum ratios of 0.8, the flexural strength increases by 63%, 75% and 102% for 1%, 1.5% and 2% carbon fiber incorporations, respectively.

There is a limit for the incorporation of carbon fibers for the 0.6 ratio, above which, contrary to expectations, the mechanical characteristics of the material do not improve overall.

The SEM images show a random grid structure for the carbon fibers. The composite displays considerable uniformity in the transition areas, creating stable mechanical cohesion, which justifies the increase in the material’s mechanical flexural strength.

Dosages with a water/gypsum ratio of 0.6 and an incorporation of carbon fibers of 1.5% display a material with a slight reduction in density, very high flexural strength, achieving an increase of up to 30%, and extremely high toughness and resistance values. This material is lighter and features characteristics which are suitable for large prefabricated construction elements in the form of panels or boards. These characteristics can be achieved, albeit with lower mechanical strength, in the case where a material with greater plasticity is required, using dosages of 2% carbon fiber and a water/gypsum ratio of 0.8. 

## 5. Patents

This investigation has resulted in the achievement of the Spanish Patent ES 2732159 B2, 13 March 2020. Herrero-del-Cura, S.; López-Zaldívar, O.; Lozano-Díez, R.V.; Hernández-Olivares, F.; Mayor-Lobo, P.L. Lightweight gypsum with powdered rubber from end-of-life tires (ELTs) and reinforced using carbon fibers, procurement procedure and use. 

## Figures and Tables

**Table 1:** Technical characteristics of the plaster used.

Mixing water	75%
Purity Index	92%
Mechanical flexural tensile strength	≥3.5 $\frac{N}{mm^{2}}$
Initial setting time (A/E = 0.8)	6–12 min
Final setting time (A/E = 0.8)	20–25 min
Whiteness index	>85%
Granulometry	Retention <1% in a 200-micron mesh sieve according to UNE standard
Water of crystallization	5.2–6.2%

**Table 2:** Physical properties and chemical analysis of the powdered rubber waste.

Chemical Analysis:	Minimum (%)	Maximum (%)
Ketone extract	10	20
NR/SR polymers	40	55
Natural rubber (NR)	21	42
Carbon black	30	38
Ashes	3	7
Sulfur	-	5
**Physical Properties**		
Apparent density	0.5 ± 0.05 g/cm³	
Water content	<0.75% in weight	
Ferromagnetic materials	<0.01% in weight	
Textile material content	<0.25% in weight	
Other impurity content	<0.25% in weight	

**Table 3:** Granulometric analysis of the powdered rubber waste.

Nominal Sieve Aperture	% Passes through Each Sieve	
	Minimum%	Maximum%
0.800	100	100
0.500	50	80
0.250	5	30
0.125	0	10
0.063	0	5

**Table 4:** Carbon fiber properties.

Type of Fiber	Density (g/m^3^)	Length (mm)	Diameter (µm)	Modulus of Elasticity (GPa)	Ultimate Elongation (%)
**Carbon**	1.95	12	100	230	2.1

**Table 5:** Name and composition of the mixes studied.

Compound	% Rubber (Weight)	Rubber (g)	% Carbon Fiber (Weight)	Carbon Fiber (g)	w/g_1_ Ratio	Water (g)	Gypsum (g)
0.6-REF	0	0	0	0	0.6	600	1000
0.6-R5	5	50	0	0	0.6	600	950
0.6-R5-C1	5	50	1	10	0.6	600	940
0.6-R5-C1.5	5	50	1.5	15	0.6	600	935
0.6-R5-C2	5	50	2	20	0.6	600	930
0.8-REF	0	0	0	0	0.8	800	1000
0.8-R5	5	50	0	0	0.8	800	950
0.8-R5-C1	5	50	1	10	0.8	800	940
0.8-R5-C1.5	5	50	1.5	15	0.8	800	935
0.8-R5-C2	5	50	2	20	0.8	800	930

**Table 6:** Shore C hardness, density, flexural strength, compressive strength and dynamic moduli of elasticity results for the different series analyzed.

Compound	Superficial Hardness (Shore C)	Density (kg/m^3^)	Flexural Strength (MPa)	Compressive Strength (MPa)	Young’s Modulus (MPa)
0.6-REF	89.57	1234.19	7.20	18.98	8961.41
0.6-R5	87.50	1175.49	6.04	13.97	7017.62
0.6-R5-C1	82.27	1140.95	11.78	13.16	6602.02
0.6-R5-C1.5	89.03	1156.69	13.12	12.63	7473.05
0.6-R5-C2	87.07	1088.68	9.40	11.15	6879.63
0.8-REF	75.10		4.47	9.93	4973.57
0.8-R5	66.47	943.88	3.38	6.03	4024.81
0.8-R5-C1	75.37	955.18	5.53	7.37	4682.46
0.8-R5-C1.5	76.13	944.27	5.92	7.77	4859.61
0.8-R5-C2	75.47	970.94	6.83	8.19	4982.98

**Table 7:** Experimental stress–strain relationship in bending tests on prismatic samples of the different mortar mixes (w/g_1_ 0.8).

w/g_1_		REF	R5	R5-C1	R5-C1.5	R5-C2
0.6	Resistance (MJ/m^3^)	0.007	0.006	0.011	0.033	0.014
	Toughness (MJ/m^3^)	0.033	0.030	0.171	0.360	0.120
0.8	Resistance (MJ/m^3^)	0.005	0.004	0.004	0.005	0.022
	Toughness (MJ/m^3^)	0.045	0.070	0.139	0.107	0.164

## Data Availability

Data sharing is not applicable to this article.
